# Intracranial arachnoid cysts: impairment of higher cognitive functions and postoperative improvement

**DOI:** 10.1186/1866-1955-5-21

**Published:** 2013-08-28

**Authors:** Priyanthi B Gjerde, Marit Schmid, Åsa Hammar, Knut Wester

**Affiliations:** 1 Department of Surgical Sciences, University of Bergen, Bergen NO-5020, Norway; 2 Department of Neurosurgery, Haukeland University Hospital, Bergen N-5021, Norway; 3 Department of Biological and Medical Psychology, University of Bergen, Jonas Liesvei 91, Bergen N-5009, Norway; 4 Division of Psychiatry, Haukeland University Hospital, Bergen N-5021, Norway

**Keywords:** Arachnoid cysts, Cognition, Cognitive control, Executive functions, Intellectual disability, Neuropsychology, Neuropsychiatry, Neurosurgery

## Abstract

**Background:**

Intracranial arachnoid cysts have been shown to yield cognitive impairment over a range of basic mental functions, and these functions normalize after surgical cyst decompression. We wanted to investigate whether such cysts may also impair executive cognitive functions, and whether surgical cyst decompression leads to an improvement.

**Methods:**

This study included 22 patients with arachnoid cysts and 13 control patients scheduled for low back surgery. All subjects were tested with Delis-Kaplan Executive Function System (D-KEFS) tests, assessing executive function 1 day before surgery and a minimum of 3 months after surgery. The data were analyzed according to scaled score computations based on raw scores provided by D-KEFS, adjusted for age, gender, and educational norms.

**Results:**

Preoperatively, the patients with cysts group performed worse than the control group in verbal knowledge, mental flexibility, inhibitory capacity, problem solving, and planning skills. Postoperatively, the patients with cysts group significantly improved performance and were no longer different from the control group in the following subtests: inhibition, inhibition/switching, letter fluency, category switching, and total switching accuracy. The patients with cysts group also significantly improved performance in color naming, category fluency, and in the Tower test, but nevertheless remained impaired at follow-up compared with the control group. The control group did not show a similar improvement, except for the Tower test. Cyst size or postoperative volume reduction did not correlate with cognitive performance or postoperative improvement. Patients with left-sided temporal cysts performed poorer than patients with right-sided cysts on a complex verbal task demanding mental flexibility.

**Conclusions:**

Arachnoid cysts seem to impair not only basic cognition, but also executive functions. Most of this impairment appears to be reversible after surgical cyst decompression. These results may have implications for future preoperative considerations for patients with intracranial arachnoid cysts.

## Background

Arachnoid cysts are a relatively common neurodevelopmental disorder with an estimated prevalence of 0.2% to 1.7% [1-5]. Arachnoid cysts have a predilection for the middle cranial fossa [6,7] and preliminary data indicate genetic mechanisms behind their formation [8,9]. Most cysts appear to be expansive, as they cause a midline shift or compression of nearby cerebral tissue or cerebrospinal fluid (CSF) compartments.

Patients with intracranial arachnoid cysts may live their entire life without any overt symptoms from the cyst, even if the cyst is large, and cognition and neurological functions appear normal. This lack of dramatic symptoms most likely reflects the brain’s ability to compensate for the presence of a slowly growing or stable expansion, and by the fact that the cyst is already present from early childhood and thus may have influenced the volume and shaping of the skull, allowing space for the cyst [10]. When clinical symptoms are present, the most frequent manifestations are headache, dizziness, and convulsive episodes. There also appears to be an association between the intracystic pressure and the strength of the reported symptoms [11].

Pressure exerted by the cyst may compromise the function of the adjacent cortex; perfusion studies support this: arachnoid cysts reduce perfusion and metabolism in surrounding cortical regions. These changes are reversible after decompression of the cyst, thus paralleling the cognitive improvements observed in the same patients [12-14].

In recent years there has been some interest in whether or not intracranial arachnoid cysts are the source of psychological or even psychiatric problems. Several disorders have been linked to cysts affecting the temporal and frontal lobes; for review, see Wester [10]. Only a few studies have examined cognition in a larger series of patients with cysts before and after surgical decompression in a systematic fashion; they all indicate that temporal cysts may impair several, mostly basic aspects of cognition and, more importantly, that cognition normalizes after surgical cyst decompression [15-22]. This normalization of cognition may occur as early as 4 hours after surgery [19].

These previous studies have mainly investigated specific subareas of cognition, such as memory, verbal perception, and visuospatial functions. Arachnoid cysts have only been studied to a limited degree in a systematic fashion to examine whether they may also affect higher cognitive functions, such as planning, cognitive flexibility, abstract thinking, inhibition, monitoring and controlling behaviour, anticipating outcomes, and adapting to changing situations.

The aim of this study is therefore to investigate whether temporal arachnoid cysts may also impair complex cognitive functions and, if so, whether surgical cyst decompression leads to an improvement. Such information may be of value when considering the indications for surgical cyst decompression.

## Methods

### Subjects

The study included 22 consecutive patients (12 males and 10 females; age range = 22 to 68 years, mean = 43 years, SD = 15.73), each with a symptomatic arachnoid cyst. Of these patients with arachnoid cysts, there were 19 cysts in the temporal fossa and three cysts affected the frontal lobe. Fourteen cysts affected the left hemisphere and eight cysts were right-sided. A detailed account of the patients is given in Table 1, including the postoperative radiological and clinical outcomes. The most common symptoms were headache and dyscognition. Only patients who spoke fluent Norwegian were included. Patients diagnosed with learning difficulties and/or ocular pathologies were excluded from this study.

**Table 1:** Individual information for the patients with cysts group, including cyst location, sidedness, Galassi type, symptoms, and postoperative neuroimaging outcome group (NOG) and clinical outcome group (COG)

**Patient number**	**Gender**	**Age (years)**	**Location**	**Side**	**Galassi type**	**Symptoms**	**NOG**	**COG**
1	F	32	Temporal	R	3	H	3	3
2	M	62	Frontal	L	-	H	1	1
3	F	61	Frontal	L	-	H	1	1
4	F	38	Temporal	R	2	H	2	2
5	M	49	Frontal	R	-	H + V	1	2
6	M	31	Temporal	L	2	H	2	2
7	M	60	Temporal	L	2	H	1	2
8	F	22	Temporal	R	1	H + D	3	2
9	F	35	Temporal	R	2	H + E	1	2
10	M	23	Temporal	L	2	H	2	1
11	M	27	Temporal	L	1	V	3	3
12	F	22	Temporal	L	1	E	2	2
13	M	48	Temporal	R	1	H	3	1
14	F	26	Temporal	L	2	H	1	1
15	M	39	Temporal	L	1	H	3	1
16	M	43	Temporal	L	2	H + D	3	1
17	M	68	Temporal	R	2	H	1	2
18	M	64	Temporal	L	2	D	2	2
19	M	43	Temporal	L	1	H + D	2	1
20	F	66	Temporal	L	1	H	1	2
21	F	41	Temporal	L	1	H + D	3	2
22	F	35	Temporal	R	1	H	2	1

Arachnoid cysts were classified according to the Galassi classification system (for temporal cysts only) [26]. The postoperative neuroimaging outcome group (NOG) and clinical outcome group (COG) were classified according to the system used by Helland and Wester [23]. E, epilepsy; COG, clinical outcome group; D, dyscognition; H, headache; L, left side; NOG, neuroimaging outcome group; R, right side; V, vertigo/dizziness.

The patients with cysts group underwent a craniotomy under general anesthesia, with the removal of cyst membranes and communication of the cyst space to the basal cisterns; for details of the surgical procedure, see Helland and Wester [23]. The postoperative neuroimaging outcome group (NOG) and clinical outcome group (COG) were classified according to the system used by Helland and Wester [23].

To rule out preoperative anxiety and postoperative relief as possible causes of poor performance in the cognitive tests or any postoperative improvement, 13 patients (nine males and four females; age range = 37 to 71 years, mean = 49.92 years, SD = 11.49), all scheduled for lumbosacral back surgery, were included in the study as controls. Patients with known pathological conditions in the head or the neck, or who had motor problems, were excluded from the control group.

Both patient groups were tested 1 day before scheduled surgery and at follow-up consultation, a minimum of 3 months after the operation.

Informed consent was obtained from all patients prior to testing, after they had received written information about the study. The study was performed in accordance with the Declaration of Helsinki of the World Medical Association. The Regional Committee for Medical Research Ethics in Norway and the Norwegian Data Inspectorate approved of the study.

### Neuropsychological test procedures

The following Delis-Kaplan Executive Function System (D-KEFS) tests were administrated: Color-Word Interference test, Verbal Fluency test, and Tower test [24]. Test conditions were standardized according to the recommendation for each test and conducted by the same examiner each time for each patient.

The D-KEFS test battery has been demonstrated to be helpful in assessing higher level cognitive functions, such as planning, problem solving, cognitive switching, inhibition, and flexibility of thinking [25].

#### Color-Word Interference test

The Color-Word Interference test is a version of a Stroop-like paradigm used to investigate inhibitory capacity. It contains the following four conditions, the first three being identical to the Stroop test: 1) color naming, where the subject is required to read color patches as fast as possible; 2) word reading, where the subject is required to read the name of the colors written in black ink; 3) inhibition, where the subject is required to name the color with which the color name is printed, thereby inhibiting the more automatic response of reading the word; and 4) inhibition/switching, the latter being almost identical to condition 3, except that when the word is presented within a frame, the subject is required to read the word, not the color with which it is printed. Basic cognitive skills measured by this test are naming of color patches (condition 1) and reading of colored words (condition 2). Executive functions tapped by this test are inhibition measured by condition 3, and inhibition and mental flexibility (set-shifting) measured by condition 4.

#### Verbal Fluency test

The Verbal Fluency test includes three conditions: 1) letter fluency, where the subject is required to generate words beginning with the letters F, A and S; 2) category fluency, where the subject is required to generate words from over-learned concepts, such as animals and boys’ names; and 3) category switching, where the subject is required to shift between naming fruit and furniture. Basic cognitive skills measured by all three conditions are vocabulary knowledge, spelling ability, and basic attention. Higher level functions, such as initiation and simultaneous processing, are tapped by all three conditions. For the specific conditions, systematic retrieval of phonemically similar lexical items (letter fluency, condition 1), rapid retrieval of multiple words from a semantic category (category fluency, condition 2), and mental flexibility (set shifting) (category switching, condition 3), are measured.

#### Tower test

The Tower test is based on the classic Tower of London task. The test consists of one board with three pegs and several beads with different colors. The examiner uses the beads and the board to present the subject with problem-solving tasks and the subject is required to build the same tower as shown on a booklet. This version of the Tower test has nine trials with increasing difficulty. Basic cognitive skills measured by this task are visual attention and visuospatial skills. Higher level functions tapped by this task are spatial planning, rule learning, and the ability to establish and maintain a cognitive set. A total achievement score (TAS) is computed, which represents a global measure of overall performance on this task.

### Data scoring and analyses

The Tower test was not administered for one patient in the control group due to sudden illness. The data were analyzed according to scaled score computations based on raw scores provided by D-KEFS, and the scaled scores were adjusted for age, gender, and educational norms.

A two-way between-groups analysis of variance (ANOVA) was conducted to assess the differences between the two groups on each condition at initial testing (test 1). For the Color-Word Interference test, data were analyzed in scaled score difference in seconds to complete each condition. For the Verbal Fluency test, data were analyzed in scaled score difference in number of words produced in 1 minute on each condition. A one-way between-groups ANOVA was conducted to assess the difference between the patient group and the control group on the Tower test. For the Tower test, a total achievement scaled score was calculated. Repeated measures of ANOVA were conducted to assess the performance of patients with cysts group and control group across two time periods, at the initial preoperative testing (test 1) and after surgery (test 2). To assess the differences between the two groups on the different conditions at test 2, ANOVA was conducted. Error analysis was conducted using independent samples *t* tests comparing the two groups on the proportion of errors made in the different conditions both at test 1 and test 2. Paired sample *t* tests were conducted to analyze the performance on each condition for the patients with cysts group and the control group, from test 1 and test 2.

Independent samples *t* tests between groups and paired sample *t* tests for each group were also computed according to clinical data, such as cyst location (frontal versus temporal cysts), cyst size (small versus large cysts), sidedness (right versus left), and postoperative neuroimaging and clinical recovery (NOG and COG), to investigate if such parameters could be associated with the patients’ test performance in test 1 and test 2.

## Results

For the patients with cysts group, a detailed account of the clinical data (cyst location, cyst sidedness, Galassi type [26] (for temporal cysts only), symptoms, and postoperative NOG and COG [23]) is presented in Table 1. All the patients with cysts exhibited evidence of successful surgical cyst decompression, with a postoperative reduction of the fluid space where the cyst had been located and/or a reduction/disappearance of the clinical symptoms. None of the patients with cysts had any postoperative complications adding to their invalidity.

In general, the patients with cysts group performed significantly worse preoperatively compared with the control group on all the subtests, except for the word reading subtest of the Color-Word Interference test (Table 2). After surgery, the patients with cysts displayed a significant improvement for all tests. The control group showed no similar improvement, except in the Tower test (Table 2).

**Table 2:** Performance of the patients with cysts group (n = 22) and the control group (n = 13) at preoperative test 1

**Test**	**Patient group**^**a**^		**Control group**^**a**^		**Statistics**^**b**^		
	**Mean**	**SD**	**Mean**	**SD**	**F**	***P***	**Eta squared**
**Color-Word Interference test, STC**							
Color naming	33.63	6.98	28.15	4.54	9.43	<0.01	0.22
Word reading	25.36	4.7	23.92	4.35	-	NS	-
Inhibition	69.14	23.45	54.62	16.46	10.03	<0.01	0.23
Inhibition/switching	81.64	25.37	69.31	13.62	9.02	<0.01	0.22
**Verbal Fluency test, NWP**							
Letter fluency	36.27	10.68	55.23	13.31	21.47	<0.01	0.39
Category fluency	37.18	8.08	44.54	6.24	8.53	<0.01	0.21
Category switching	13.27	2.86	17.92	3.5	19.71	<0.01	0.37
Total switching accuracy	12.55	2.94	17	3.39	17.91	<0.01	0.35
**Tower test, TAS**							
Total achievement score	16.73	2.68	24.17	3.74	50.09	<0.01	0.61

^a^Values for means and SDs are reported in raw scores; ^b^statistics reported (F, *P*, eta squared) are according to scaled scores. NS, no significant difference between the groups; NWP, number of words produced in 60 seconds; STC, seconds to complete trial; TAS, total achievement score.

### Performance at preoperative test 1

#### Color-Word Interference test

When the results for the dependent variables were considered separately and corrected for multiple comparisons, the patients with cysts group performed significantly worse than the control group, using more time on condition 1 (color naming), condition 3 (inhibition), and condition 4 (inhibition/switching) (Table 2). The patients with cysts group also made significantly more errors (mean = 3.27, SD = 1.93) than the control group (mean = 1.54, SD = 1.13) on condition 4 (inhibition/switching); *t*(33) = 3.36, *P* = 0.002.

#### Verbal Fluency test

The main effect of group on the combined dependent variables was statistically significant, showing a significant difference between groups in test scores. The patients with cysts group performed significantly worse than the control group on all four conditions: letter fluency, category fluency, category switching, and total switching accuracy (Table 2).

When the results for the dependent variables were considered separately and corrected for multiple comparisons, the patients with cysts group made significantly more set-loss errors, that is, when a word not belonging to the specific category is produced (mean = 0.32, SD = 0.65) than the control group (mean = 0.0, SD = 0.0) on the letter fluency condition; *t*(21) = 2.31, *P* = 0.031. The patients with cysts group also made significantly more repetition errors (mean = 0.63, SD = 1.22) than the control group (mean = 0.0, SD = 0.0) on the letter fluency condition; *t*(21) = 2.45, *P* = 0. 023. The patients with cysts group made significantly more set-loss errors (mean = 1.0, SD = 0.87) than the control group (mean = 0.39, SD = 0.65) on the category switching condition; *t*(33) = 2.20, *P* = 0.024.

#### Tower test

In general, the patients with cysts group performed worse than the control group on the preoperative test (Table 2), with significantly more errors (mean = 1.09, SD = 2.04) than the control group (mean = 0.08, SD = 0.29) on this test; *t*(22.5) = 2.27, *P* = 0.033.

In sum, preoperative test performance of the patients with cysts group was worse than the control group on measures of higher level functions, such as spatial planning, rule learning, and the ability to establish and maintain a cognitive set.

### Longitudinal data, performance from test 1 to test 2

#### Color-Word Interference test

**Figure 1:**
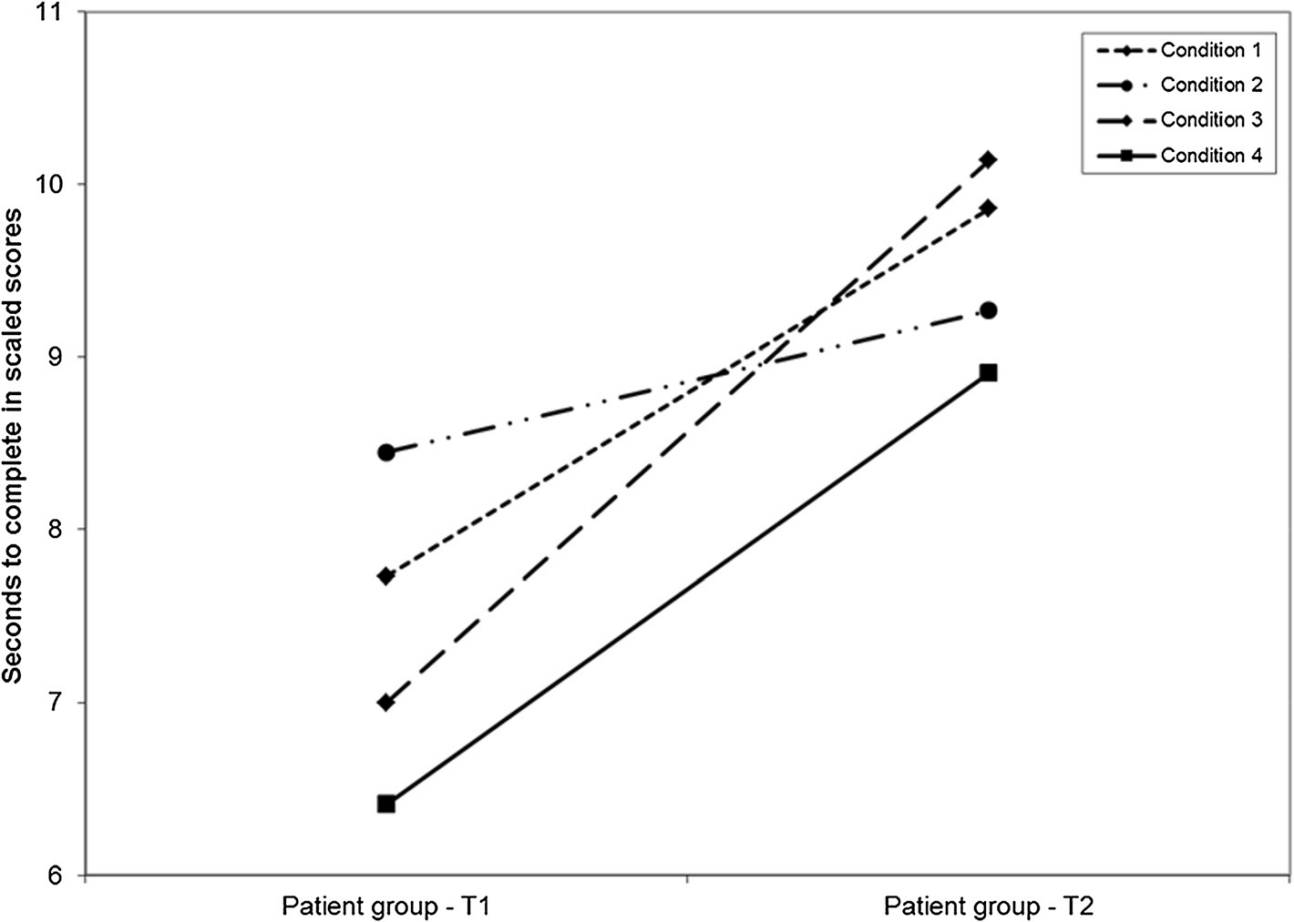
**Performance of the patients with cysts group from test 1 to test 2 on the Color-Word Interference test.** The patients with cysts group showed a significantly improved postoperative test performance on all conditions, except the word reading condition. Condition 1, color naming; condition 2, word reading; condition 3, inhibition; condition 4, inhibition/switching.

The performance of patients with cysts group improved significantly on the inhibition condition from test 1 (mean = 7.00, SD = 4.07) to test 2 (mean = 10.14, SD = 3.11); *t*(21) = −4.08, *P* = 0.001 (two-tailed), and were no longer significantly different from the control group. The patients with cysts group also improved significantly on the inhibition/switching condition from test 1 (mean = 6.41, SD = 3.63) to test 2 (mean = 8.91, SD = 2.67); *t*(21) = −4.88, *P* = 0.000 (two-tailed), and were no longer different from the control group (Figure 1; Table 3). A similar improvement at postoperative test 2 was not seen for the control group (Table 3). The performance of patients with cysts group at test 2 on the word reading condition did not reach statistical significance (Figure 1), and there was still a significant difference between the performance of the two groups in the color naming condition, even when the patients with cysts group improved significantly (Figure 1; Table 3).

**Table 3:** Performance of the patients with cysts group (n = 22) and the control group (n = 13) at postoperative test 2

**Test**	**Patient group**^**a**^		**Control group**^**a**^		**Statistics**^**b**^		
	**Mean**	**SD**	**Mean**	**SD**	**F**	***P***	**Eta squared**
**Color-Word Interference test, STC**							
Color naming	29.46	4.5	26.15	3.56	5.2	<0.05	0.14
Word reading	25.82	12.99	22.85	3.91	-	NS	-
Inhibition	56.41	14.58	53.69	17.38	-	NS	-
Inhibition/switching	68.96	14.71	68.85	13.19	-	NS	-
**Verbal Fluency test, NWP**							
Letter fluency	55.09	10.37	58.15	12.48	-	NS	-
Category fluency	48.82	8.61	43.31	5.34	4.39	<0.05	0.21
Category switching	18.59	3.28	19.69	2.81	-	NS	-
Total switching accuracy	17.46	3.19	18.69	2.81	-	NS	-
**Tower test, TAS**							
Total achievement score	20.73	3.51	27.58	2.15	35.43	<0.01	0.53

^a^Values for means and SDs are reported in raw scores; ^b^statistics reported (F, *P*, eta squared) are according to scaled scores. NS, no significant difference between the groups; NWP, number of words produced in 60 seconds; STC, seconds to complete trial; TAS, total achievement score.

There was a significant difference between the patients with cysts group and the control group in test scores from test 1 to test 2, with a significant main effect of group; F(1.33) = 7.84, *P* = 0.008, partial eta squared = 0.92. All the conditions of the test differed significantly, with a main effect for condition; Wilks’ lambda = 0.77, F(3.31) = 3.15, *P* = 0.039, partial eta squared = 0.23). Further, the scores in the different conditions were statistically different from test 1 to test 2, and there was a main effect of time; Wilks’ lambda = 0.59, F(1.33) = 22.58, *P* = 0.000, partial eta squared = 0.41. In addition, the two groups performed with significant difference from test 1 to test 2, and the two-way interaction of time and group was significant; Wilks’ lambda = 0.89, F(1.33) = 9.79, *P* = 0.004, partial eta squared = 0.23. Moreover, the two groups performed with significant difference on the various conditions from test 1 to test 2, and the three-way interaction of condition, time, and group was significant; Wilks’ lambda = 0.70, F(1.31) = 4.44, *P* = 0.010, partial eta squared = 0.30.

Even though the patients with cysts group performed poorer than the control group on the color naming condition, they still performed significantly better on test 2 (mean = 9.86, SD = 2.59) than on test 1 (mean = 7.73, SD = 3.24) for this particular condition; *t*(21) = −4.72, *P* = 0.000 (two-tailed). A similar improvement was also found for the control group as they performed better on the color naming condition from test 1 (mean = 10.85, SD = 2.19) to test 2 (mean = 11.69, SD = 1.65) on the color naming condition; *t*(12) = −2.86, *P* = 0.014 (two-tailed).

In sum, the patients with cysts group improved their test performance significantly after surgery compared with the control group, and were no longer different from the control group in the higher level function inhibition.

#### Verbal Fluency test

**Figure 2:**
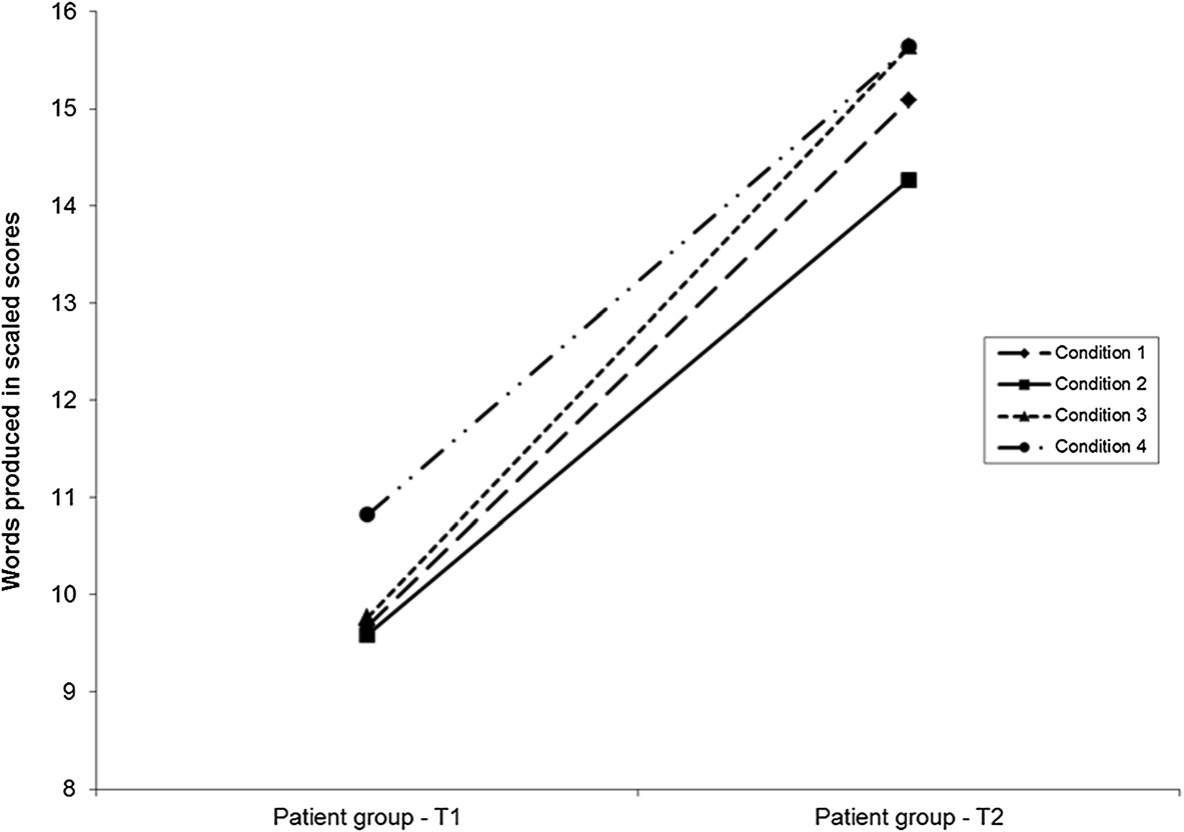
**Performance of the patients with cysts group from test 1 to test 2 on the Verbal Fluency test.** The patients with cysts group showed a significantly improved postoperative test performance for all the conditions. Condition 1, letter fluency; condition 2, category fluency; condition 3, category switching; condition 4, total switching accuracy.

The patients with cysts group significantly improved test performance from test 1 to test 2 in for all four conditions (Figure 2). However, the patients with cysts group still performed significantly poorer than the control group on the category fluency condition (Table 3).

There was a difference between groups in test scores from test 1 to test 2, with a significant main effect of group; F(1.33) = 11.69, *P* = 0.002, partial eta squared = 0.26. The test conditions also differed significantly, as there was a main effect for condition; Wilks’ lambda = 0.44, F(3.31) = 12.73, *P* = 0.000, partial eta squared = 0.55. In addition, the scores in the various conditions differed significantly from test 1 to test 2, with a main effect of time; Wilks’ lambda = 0.42, F(1.33) = 45.18, *P* = 0.000, partial eta squared = 0.59. Moreover, the two groups differed significantly in their performance from test 1 to test 2, and the two-way interaction of time and group was significant; Wilks’ Lambda = 0.61, F(1.33) = 21.36, *P* = 0.000, partial eta squared = 0.39. Also, the various conditions differed significantly from test 1 to test 2, and the two-way interaction of time and condition was significant; Wilks’ lambda = 0.61, F(3.31) = 6.49, *P* = 0.002, partial eta squared = 0.39. Furthermore, there was a significant difference between groups and test performance, and the two-way interaction of condition and group was significant; Wilks’ lambda = 0.69, F(3.31) = 4.60, *P* = 0.009, partial eta squared = 0.31. Finally, the groups performed with significant difference on the conditions from test 1 to test 2, and the three-way interaction of time, condition, and group was significant; Wilks’ lambda = 0.75, F(3.31) = 3.35, *P* = 0.032, partial eta squared = 0.25.

The patients with cysts group performed significantly better on the letter fluency condition at postoperative test 2 (mean = 15.09, SD = 2.84) than preoperative test 1 (mean = 9.68, SD = 3.26); *t*(21) = −8.50, *P* = 0.000 (two-tailed). The control group also performed significantly better on the letter fluency condition from test 1 (mean = 15.31, SD = 3.82) to test 2 (mean = 16.07, SD = 3.73); *t*(12) = −4.63, *P* = 0.001 (two-tailed). The patients with cysts group also improved postoperatively in the category fluency condition from test 1 (mean = 9.59, SD = 3.11) to test 2 (mean = 14.27, SD = 3.15); *t*(21) = −8.31, *P* = 0.000 (two-tailed); category switching condition from test 1 (mean = 9.77, SD = 3.38) to test 2 (mean = 15.64, SD = 3.29); *t*(21) = −6.31, *P* = 0.000 (two-tailed); and total switching accuracy from test 1 (mean = 10.82, SD = 2.86) to test 2 (mean = 15.64, SD = 2.97); *t*(21) = −6.15, *P* = 0.000 (two-tailed) (Figure 2). The control group did not show a similar improvement in category fluency, category switching, and total switching accuracy.

In sum, the patients with cysts group showed a significant postoperative improvement on the higher level functions of initiation and simultaneous processing compared with the control group, which did not show any significant improvement of these higher level cognitive functions.

#### Tower test

The patients with cysts group, which had a significantly poorer performance at test 1 than the control group, improved postoperatively from test 1 (mean = 10.18, SD = 1.89) to test 2 (mean = 13.05, SD = 2.57); *t*(21) = −6.33, *P* = 0.000 (two-tailed). However, the control group also performed significantly better from test 1 (mean = 15.50, SD = 2.43) to test 2 (mean = 17.75, SD = 1.22); *t*(11) = −5.48, *P* = 0.000 (two-tailed). Despite the postoperative improvement, the patients with cysts group still performed significantly poorer than the control group on test 2 (Table 3). The two groups did not differ in performance on the Tower test from test 1 to test 2, since the two-way interaction of time and group was not significant.

In sum, even though the patients with cysts group did improve their performance after surgery, they still performed significantly poorer than the control group, as the control group also improved their performance from test 1 to test 2 on measures of higher level functions, such as spatial planning, rule learning, and the ability to establish and maintain a cognitive set.

#### Frontal versus temporal cyst location

##### Color-Word Interference test

There was no significant difference between patients with frontal cysts (n = 3) and patients with temporal cysts (n = 19) at test 1 or test 2. However, there was a difference between the two groups concerning improvement from test 1 to test 2. The patients with temporal cysts significantly improved performance on all conditions of the Color-Word Interference test. This pattern was not seen for the patients with frontal cysts.

##### Verbal Fluency test

Patients with the frontal cysts performed poorer compared with patients with temporal cysts on the category fluency condition, the category switching condition, and the category total switching accuracy condition at test 2, but no differences were found at test 1. Further, both groups showed a significant postoperative improvement on all conditions of the Verbal Fluency test from test 1 to test 2, except the patients with frontal cysts, who did not significantly improve performance on the category switching condition.

##### Tower test

There was no difference in performance between the patients with frontal cysts and temporal cysts on the Tower test at test 1 or test 2, and both groups showed a significant postoperative improvement.

#### Temporal cysts, sidedness, and size

Patients with left-sided temporal cysts (n = 12) performed significantly poorer than the patients with right-sided cysts (n = 7) on the inhibition/switching (mental flexibility) condition of the Color -Word Interference test at both test 1 (*P* = 0.006 (two-tailed)) and test 2 (*P* = 0.021 (two-tailed)). There was no difference in performance between the two groups for the other executive functions measured. There was no difference between patients with small cysts (Galassi type 1; n = 9) compared with patients with larger cysts (Galassi type 2 to 3; n = 10).

#### Neuroimaging outcome group (NOG) and clinical outcome group (COG)

There was no significant difference between patients with a significant radiological improvement (NOG 1 and 2; n = 15) and patients with a minor or no radiological improvement (NOG 3 and 4; n = 7) in executive functions at test 1 or test 2.

There was no significant difference in postoperative executive functioning at test 2 between the patients exhibiting subjective improvement (COG 1 and 2; n = 20) and patients without a clinical improvement (COG 3; n = 2) in the Color-Word Interference test and the Verbal Fluency test. However, the patients without clinical improvement (COG 3) performed significantly poorer compared with the other patients on the Tower test at both test 1 and test 2.

## Discussion

This study demonstrates that intracranial arachnoid cysts may cause deficits in higher level executive functions, such as inhibition, cognitive flexibility, rule learning, planning, problem solving, and initiating tasks. Furthermore, the present results show for the first time that patients significantly improve their ability to perform executive functions after surgical decompression of the cyst. The study also adds to the substantiation that cysts may hamper cognitive dysfunction involving more basic cognitive skills, as shown in previous studies [15-22,27].

There are reasons to believe that preoperative cognitive impairment and postoperative improvement of the patients with cysts reflect real effects of the cyst and surgical cyst decompression. To rule out preoperative anxiety and postoperative relief as possible causes for any preoperative impairment and postoperative improvement, respectively, we used patients undergoing back surgery as controls. It is conceivable that such emotional sources of error will be more pronounced in patients with cysts than in patients undergoing less dramatic, extra-cranial procedures. We believe, however, that it will be difficult to find more suitable controls, since any other patients with intracranial conditions may well have cognitive impairment and would therefore not be suited. Since the control group did not show a similar overall postoperative improvement as the patients with cysts group did, except in the Tower test, it also seems fair to rule out a general learning effect of the test situation as the cause of the postoperative improvement in the patients with cysts group.

Neuroimaging studies have shown that a cyst may reduce the perfusion and metabolism in the surrounding cortical regions, and that these changes are reversed after cyst decompression, thus paralleling the clinical and cognitive improvements seen in the same patients [12-14]. Similarly, we assume that this normalized perfusion is the cause of the postoperative improvement observed in this study.

Since arachnoid cysts are congenital malformations, it is a common misapprehension that such cysts in adults have affected the contiguous cerebral tissue for so long a time that brain damage has occurred and that the clinical manifestations might be permanent, and therefore refractory to any form of recuperation. The relatively rapid restoration of executive functions and other cognitive functions after surgical decompression in this and previous studies, demonstrates that this is not the case. Rather than causing a permanent destruction of the surrounding brain tissue, it appears more likely that arachnoid cysts cause a reversible suppression of brain functions, as suggested by Raeder *et al*. [16]. Basic as well as executive cognitive functions are vital. The fact that arachnoid cysts cause cognitive deficits, and that this dyscognition normalizes after cyst decompression, should in our view be taken into consideration when setting the indication for surgical cyst decompression.

However, not all functions improved to the same extent. For two of the conditions, category fluency (condition 2, Verbal Fluency test) and color naming (condition 1, Color-Word Interference test), and the Tower test, the patients with cysts group failed to reach the same postoperative score levels as the control group, even though they showed significant improvement from test 1 to test 2. This may indicate that some functions have been permanently affected by the cyst or, alternatively, that these functions require more time to recuperate. It would therefore be interesting to have a longer follow-up period to investigate whether the impairment is completely reversible following surgery.

Our group has previously demonstrated that there is no association between cyst volume reduction and clinical improvement [23]. Before and after birth, the developing neurocranium is molded after its content. Thus, the presence of a congenital arachnoid cyst will create a surplus intracranial space during the growth of the skull, such as the enlarged bony middle fossa often seen in patients with temporal fossa cysts. A large cyst may create a large surplus space, too large for the expanding brain to fill all the vacant space after surgical decompression. This phenomenon may explain not only the lack of association between clinical and neuroimaging outcomes, but also the lack of association between cyst size and cognitive impairment as observed in the present study. For visuospatial cognition, our group has previously demonstrated a similar lack of association between cyst size and maze learning [22].

Whether the observed effect is caused by affection of the frontal lobe or the temporal lobe remains uncertain, since a temporal arachnoid cyst, primarily located in the middle cranial fossa, may well also affect the neighboring frontal lobe. The frontal lobe is assumed to harbor higher, executive functions; for review, see Brower and Price [28], and Duffy and Campbell [29].

Whatever the reasons are, the fact that the patients with cysts did not improve indiscriminately on all tasks indicates a certain specificity of the test battery we used. The observation that there was a difference between patients with left and right temporal cysts, with the former performing worse on tests involving complex verbal tasks (the inhibition/switching condition of the Color-Word Interference test), also indicates a certain specificity of the test battery.

The present norm is that surgical treatment of patients with intracranial cysts is concluded on the basis of radiological evidence and strong symptomatic manifestations that can be coupled to the cyst location. In patients that have placid and unspecific symptoms only, there are diverging views on whether or not they should undergo surgery. The question is whether specific neuropsychological tests should be used regularly in clinical practice to disclose even subclinical cognitive problems. We believe they should.

## Conclusions

Our results imply that intracranial arachnoid cysts can cause a reversible impairment of executive functions, and that surgical decompression normalizes these aspects of cognition. These results may have implications for future preoperative considerations in patients with cysts.

### Ethical approval

The Regional Committee for Medical Research Ethics in Norway and the Norwegian Data Inspectorate approved of the study.

## Abbreviations

ANOVA: Analysis of variance; COG: Clinical outcome group; CSF: Cerebrospinal Fluid; D-KEFS: Delis-Kaplan executive function system; NOG: Neuroimaging outcome group; TAS: Total achievement score.

## Competing interests

The authors declare that they have no financial or other competing interests. They received no funding.

## Authors’ contributions

PG participated in the design of the study, conducted the neuropsychological testing of all the included individuals, analyzed the data, and wrote the manuscript. ÅH designed the study in collaboration with KW and PG, and was responsible for the neuropsychological assessment and interpretation of behavioral data. MS performed the statistical analysis and participated in the writing of the manuscript. KW conceived and designed the study in collaboration with ÅH and PG, performed all the surgical decompressions, and wrote and revised the manuscript. All authors read and approved the final manuscript.
